# Overview of a chemoresponse assay in ovarian cancer

**DOI:** 10.1007/s12094-014-1192-8

**Published:** 2014-07-02

**Authors:** E. C. Grendys, J. V. Fiorica, J. W. Orr, R. Holloway, D. Wang, C. Tian, J. K. Chan, T. J. Herzog

**Affiliations:** 1 Florida Gynecologic Oncology and Regional Cancer Center, 8931 Colonial Center Drive, Fort Myers, FL 33905 USA; 2 First Physicians Group Gyn Onc, 1888 Hillview St., Sarasota, FL 34239 USA; 3 Florida Hospital Cancer Institute, 2501 N. Orange Avenue, Orlando, FL 32804 USA; 4 Precision Therapeutics, 2516 Jane Street, Pittsburgh, PA 15203 USA; 5 Gynecologic Oncology, Palo Alto Medical Foundation/Research Institute, Sutter Cancer Research Consortium, 3838 California Street #404, San Francisco, CA 94115 USA; 6 University of Cincinnati Cancer Institute, University of Cincinnati Medical Center, 222 Piedmont Ave. Suite 4100, Cincinnati, OH 45219 USA

**Keywords:** Chemoresponse assay, Chemo sensitivity and resistance assay, Ovarian cancer, Chemotherapy, Personalized medicine, Individualized therapy

## Abstract

The objective of this review is to summarize recent scientific and medical literature regarding chemoresponse assays or chemotherapy sensitivity and resistance assays (CSRAs), specifically as applied to epithelial ovarian cancer. A total of sixty-seven articles, identified through PubMed using the key words “in vitro chemoresponse assay,” “chemo sensitivity resistance assay,” “ATP,” “HDRA,” “EDR,” “MiCK,” and “ChemoFx,” were reviewed. Recent publications on marker validation, including relevant clinical trial designs, were also included. Recent CSRA research and clinical studies are outlined in this review. Published findings demonstrate benefits regarding patient outcome with respect to recent CSRAs. Specifically, analytical and clinical validations, as well as clinical utility and economic benefit, of the most common clinically used CSRA in the United States support its use to aid in making effective, individualized clinical treatment selections for patients with ovarian cancer.

## Ovarian cancer

Ovarian cancer is the most lethal and second most common gynecologic malignancy in the United States, with an estimated 21,980 new cases and 14,270 deaths expected in 2014 [1]. First progression typically occurs within 18 months, and overall survival (OS) is typically <4 years [2–5]. Most patients are present with advanced disease, and the current standard of care in the primary setting is surgical debulking followed by platinum-based chemotherapy.

Ovarian cancer is a heterogeneous disease with respect to histopathology, molecular biology, and clinical outcome, suggesting that a single standard treatment is unlikely to benefit all patients. Histologically, most ovarian cancer arises from the distal fallopian tube or ovarian surface, and the majority of these epithelial ovarian cancers (EOC) are serous/papillary pathological subtype, followed by endometrioid, mucinous, clear cell, and undifferentiated. These different subtypes—together with other clinical factors including age, performance status, FIGO stage, differentiation, ascites presence, and surgical debulking status—are important prognostic factors. Recent studies examined ovarian cancer heterogeneity at the molecular level. The Cancer Genome Atlas project found that more than 30 growth-stimulating genes were altered across different ovarian cancer subtypes. These alterations included: PI3K pathway activation, BRCA1 or BRCA2 mutations, other DNA repair defects and varied expression status of ER, cyclin E2, and kit [6]. This molecular heterogeneity may be linked to clinical heterogeneity, such as histological subtype presentation, disease prognosis, and chemotherapy efficacy.

Carboplatin/paclitaxel has been widely accepted as the standard of care in treating primary EOC for nearly two decades [2–5]. Multiple alternate regimens have been investigated, most of them based on the platinum/taxane standard, but augmented with additional chemotherapies and/or altered sequencing (Table 1) [2–5]. Many studies have randomized patients across various regimens in an effort to identify regimens superior to the carboplatin/paclitaxel standard. These studies have consistently demonstrated remarkably similar progression-free survival (PFS) and OS between the standard of care and the various alternates, highlighting the therapeutic equivalence of the various regimens and the associated empiric treatment ambiguity.

**Table 1:** Patient characteristics of control arm and assay-informed arm cohorts

Patient characteristics		Control arm				Assay-informed arm
		du Bois JNCI 2003	Pfisterer JNCI 2006	du Bois JCO 2006	Bookman JCO 2009	Herzog AJOG 2010
Number of patients		783	1,308	1,282	4,312	192
Median age		57	60	59	59	59
Pathological subtype	Serous/papillary	70 %	71 %	73 %	80 %	71 %
	Other	30 %	29 %	27 %	20 %	29 %
FIGO stage distribution	Stage I		<1 %	<1 %		
	Stage II	8 %	9 %	9 %		
	Stage III	75 %	74 %	74 %	85 %	84 %
	Stage IV	17 %	17 %	17 %	15 %	16 %
Debulking status	Optimal	63 %	67 %	68 %	68 %	52 %
	Sub-optimal	37 %	33 %	32 %	32 %	48 %
Treatment regimens		CP CisP	CP CPT	CP CPE	CP CPG CPD CT/CP CG/CP	CP CPG CisP CT Other

*C* carboplatin, *P* paclitaxel, *G* gemcitabine, *D* doxorubicin, *E* epirubicin, *T* topotecan, *Cis* cisplatin

For patients with recurrent, persistent, or progressive disease, chemotherapy choice is currently based, in part, on the duration and type of response to initial therapy. For platinum-sensitive disease [progression-free interval (PFI) ≥6 months from the end of platinum/taxane therapy], a platinum-based combination regimen is usually empirically selected. For platinum-resistant disease (PFI <6 months), physicians empirically select from an array of non-platinum regimens, including pegylated liposomal doxorubicin (PLD), topotecan, gemcitabine, etoposide, taxanes, and targeted therapies, all of which have been evaluated and demonstrated to be clinically equivalent and acceptable for use in this patient population [7].

While marker identification and development in ovarian cancer is generally limited to early detection, monitoring progression, or detecting recurrence, there are some encouraging preliminary studies linking markers with drug response, thereby demonstrating early potential for informing effective individualized chemotherapy selection. For example, expression of Copper importers/exporters, ERCC1, Tau, GST-Pi, MLH1, and XIAP, and mutations of MLH1, BRCA1/BRCA2, and p53 have been linked to platinum response, and expression of TGFBI, Survivin, and mutation of tubulin are associated with response to paclitaxel [8–21]. However, none of these biomarkers have demonstrated sufficient clinical validation required to inform clinical treatment decisions.

## Chemoresponse assays: a panel of treatment response markers

The National Institutes of Health (NIH) Biomarkers Definitions Working Group, which includes leaders in the field from the Food and Drug Administration (FDA), NIH, academia and industry, defines a marker as “a characteristic that is objectively measured and evaluated as an indicator of normal biologic processes, pathogenic processes, or pharmacologic responses to a therapeutic intervention” [22]. A marker was similarly described by Hayes et al. as “a molecular, cellular, tissue, or process-based alteration that provides indication of current, or more importantly, future behavior of a cancer” [23].

A chemoresponse assay reports a panel of markers characterizing a tumor’s response to multiple chemotherapy agents. Each of the multiple chemotherapy assay results reported is a singular marker associated with a distinct treatment. Such assays provide tumor response information aimed at aiding in the selection of effective, individualized treatment regimens. Chemoresponse assays provide the same utility as other treatment markers that are associated with patient outcome when the given marker’s associated treatment is clinically administered (e.g., KRAS and cetuximab/panitumumab, EGFR and erlotinib, Her2 and trastuzumab). Chemoresponse assays are generally based on phenotypic rather than molecular characterization, thus enabling assays to simultaneously report multiple treatment markers, each associated with a distinct treatment, for a given patient [24].

The concept of a chemoresponse assay or chemotherapy sensitivity and resistance assay (CSRA), originated in the 1950s [25]. There are different types of CSRAs such as the adenosine triphosphate (ATP) assay, human tumor cloning assay (HTCA), methylthiazolyl-diphenyl-tetrazolium bromide (MTT) assay, extreme drug resistance (EDR) assay, as well as assays utilizing drug-induced apoptosis as the end point [24, 26, 27]. A high-impact HTCA study published in 1978 was followed by decades of research from various academic groups and a few commercial entities with mixed results [28–31]. While other CSRA reviews have been published previously [24, 26], this review will focus on progress made during the most recent decade.

The value of CSRAs to inform effective treatment selection for individual patients remains a compelling clinical question and a highly debated topic among oncologists. While the advantages and disadvantages of various CSRA methods have been published, clinical validations demonstrating association of assay results with patient outcomes through prospective studies have the most value. Several CSRA clinical validations have been reported recently. A prospective histoculture drug response assay (HDRA) study in advanced EOC patients (*n* = 104) treated with carboplatin and paclitaxel after cytoreductive surgery demonstrated a lower recurrence rate and extended PFS, both of which were statistically significant, in the HDRA-sensitive group as compared to the HDRA-resistant group [32]. Another prospective study, utilizing an ATP-based chemoresponse assay, evaluated response rate and PFS in platinum-resistant recurrent EOC patients (*n* = 180) randomized to assay-directed or physician’s empiric therapy choice, demonstrating trends for improved response rate and PFS for assay-informed treatment [33]. And, finally, a prospective study of 113 recurrent EOC patients showed that patients whose treatment was determined by an ATP-based chemoresponse assay had statistically longer PFS and higher overall response rates compared with patients receiving physician’s-choice therapy [34]. Numerous retrospective data have also been published in the past decade, with the majority reporting statistically significant associations between assay results and clinical outcomes [35–37]. The results from these various studies reasonably demonstrate the clinical potential of chemoresponse assays in both primary and recurrent EOC.

Although several chemoresponse assays’ clinical validity and clinical utility have been evaluated in clinical trials, there are currently only two assays commercially available in the United States: the Microculture-Kinetic (MiCK) assay (DiaTech Oncology, Franklin, TN) and the ChemoFx^®^ assay (Precision Therapeutics, Inc., Pittsburgh, PA). The MiCK assay is based on drug-induced apoptosis and was originally developed in hematologic malignancies where it was noted that chemotherapeutic drugs have the ability to rapidly induce apoptosis in tumor cells in short-term culture. The assay was later applied to solid tumors, including breast, lung, and gynecologic malignancies [27, 38–41]. Clinical validation of the MiCK assay in 73 ovarian cancer patients demonstrated that clinical treatment with the assay-indicated “best” chemotherapy is an independent predictor of OS in multivariate analysis of chemotherapy-naïve stage III or IV primary ovarian cancer patients [27]. A clinical utility study of 44 cancer patients showed that oncologists used the MiCK assay to determine chemotherapy selection in 28 patients (64 %) and did not use the assay in treatment selection for the other 16 patients (36 %). The median OS was 10.1 months for the assay-informed patients vs. 4.1 months for the assay-uninformed patients (*p* = 0.02). However, the 44 tumors in the study included a variety of tissue types, such as breast and non-small lung cancers; only two ovarian cancer tumors were included in the study [38]. Therefore, the clinical validation and clinical utility of this assay in ovarian cancer requires further investigation. The ChemoFx chemoresponse assay has been extensively evaluated in patients with ovarian cancer and will be the focus of the remainder of this review.

## ChemoFx

### Assay process

**Fig. 1:**
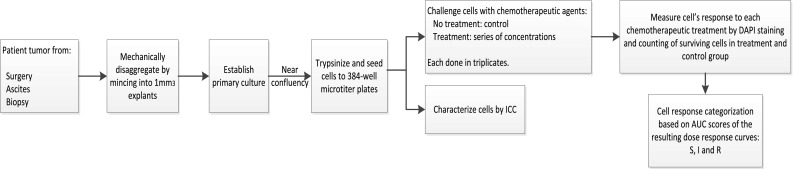
ChemoFx assay process. *ICC* immunocytochemistry, *AUC* area under curve, *S* sensitive, *IS* intermediate sensitive, *R* resistant

ChemoFx is a chemoresponse assay that characterizes both the sensitivity and resistance of a patient’s tumor to various physician-selected, clinically applicable chemotherapy treatments. It quantifies chemotherapy effect by direct visualization and enumeration of live cells following exposure to these treatments. The assay is performed in a Clinical Laboratory Improvement Amendments (CLIA) and New York State Department of Health (NYSDOH) approved facility. The assay procedure is illustrated in Fig. 1 and has been previously reported [42–46].

In contrast to other CSRAs, this chemoresponse assay is characterized by several features that make it more reproducible and clinically accessible.

- The assay uniquely insures that tumor cells are proliferating prior to chemotherapy exposure, thereby measuring treatment efficacy at halting proliferation and/or killing tumor cells. This approach accommodates the cell cycle-specific, cytostatic, and cytotoxic natures of various chemotherapies.
- The assay’s primary culture process is optimized to generate sufficient proliferating tumor cells for testing. As a result, 9 out of 10 ovarian cancer samples meeting the incoming sample criteria, such as sufficient sample size and absence of microorganism contamination, are successfully reported.
- The culture process favors epithelial tumor cell proliferation and incorporates an immunocytochemistry (ICC) step to insure that the majority of cells tested are epithelial.
- The assay process is highly automated. Cell seeding into microtiter plates, serial treatment dilution and application, cell fixation, fluorescence staining, as well as cell enumeration are performed using automated liquid handling robotics, computer-assisted microscopy, and automated cell-counting algorithms and software. The automated process strongly contributes to the high throughput and reproducibility of the assay [43].
- Lastly, this assay requires significantly less tissue (a minimum of 35 mm^3^), as compared to historical assays. Tumor tissue from surgical excision, biopsy, or paracentesis is compatible, making the assay highly clinically accessible [42].

### Analytical and clinical validation

The analytical performance of this assay has been previously reported [42, 43, 46]. Heinzman et al. demonstrated a coefficient of variation (CoV) of 3.6–4.6 % for SK-OV-3 cells treated with doxorubicin, across three operators and 9 days [42]. In addition to variability across operators and days, process variability due to inter- and intraday stability of the chemotherapeutic treatments has also been reported [46]. The assay has demonstrated the necessary analytical performance characteristics required by both CLIA and NYSDOH.

Clinical validation of this assay has been the subject of numerous studies in EOC. Gallion et al. demonstrated the association of assay response with PFS in 256 EOC patients. In patients with either an exact or partial match between treatments assayed and those that were clinically administered, the hazard ratio (HR) for progression in patients clinically treated with an assay-resistant (R) vs. assay-sensitive (S) treatment was 2.1 (95 % CI 1.2–3.6, *p* = 0.01). In the subset of 135 patients with an exact match, the HR for progression in patients clinically administered an assay-R vs. assay-S treatment was 2.9 (95 % CI 1.4–6.3, *p* < 0.01). The median PFS for patients treated with R therapies was 9 and 14 months for those treated with intermediate sensitive (IS) therapies. Furthermore, at the time of study completion with a median follow-up time of 14.6 months, 60 % of patients treated with S therapies remained relapse-free [45].

Herzog et al. subsequently reported an association between assay response and OS in 192 patients with advanced EOC following first-line platinum-based chemotherapy. Median OS was 72.5, 48.6, and 28.2 months for patients who were treated with agents reported as S, IS, and R, respectively (HR = 0.7, 95 % CI 0.50–0.97, *p* = 0.03). Multivariate Cox regression analysis demonstrated that the assay prediction of response to platinum agents was a predictor of OS independent of other prognostic factors of stage, age, and optimal debulking (HR = 0.68, 95 % CI 0.49–0.95, *p* = 0.023) [44].

In another more recent observational study of 276 women with FIGO stage III-IV EOC cancer uniformly treated with first-line carboplatin-/paclitaxel-based therapy, patients with assay-R results for carboplatin were at increased risk of disease progression (as defined by PFS) compared with patients with S or IS assay results (HR = 1.87, 95 % CI 1.29–2.70, *p* = 0.0009); these results were consistent after controlling for clinical covariates (HR = 1.71, 95 % CI 1.12–2.62, *p* = 0.013). Median PFS for patients who were assay-R to carboplatin was 11.8 vs. 16.6 months for assay-IS and assay-S patients. This study demonstrates that assay resistance to carboplatin is associated with reduced PFS in EOC patients treated with standard of care carboplatin/paclitaxel, supporting the assay’s ability to identify platinum-resistant patients. Furthermore, of those patients who were resistant to carboplatin in vitro, 59 % of them displayed assay sensitivity (S or IS) to at least one other agent [48].

Finally, a prospective study of 262 women with recurrent or persistent EOC reported that patients treated with an assay-S regimen experienced significantly improved PFS (HR = 0.67, 95 % CI 0.50–0.91, *p* = 0.009) and OS (HR = 0.61, 95 % CI 0.41–0.89, *p* = 0.010) compared with those treated with assay-IS or assay-R regimens, resulting in a 14-month improvement in median OS. Assay-PFS association was consistent in both platinum-sensitive and platinum-resistant tumors (HR: 0.71 and 0.66, respectively) and was independent of other covariates (HR = 0.66, 95 % CI 0.47–0.94, *p* = 0.020). Moreover, the results indicated that more than 50 % of the patients had at least one S therapy identified by the assay, whereas only 25 % of them were empirically treated with an S drug, suggesting that the number of patients potentially experiencing improved OS may more than double when physicians reference the assay [49].

A further analysis of the 262 recurrent or persistent EOC patients reported by Rutherford et al. [49] was presented at the 2013 European Cancer Organization (ECCO) Biennial Meeting and addressed the assay’s ability to function as a predictive marker [50]. A prognostic assay identifies patients likely to respond/not respond to (any) therapy, while a predictive assay identifies a patient's likely response to specific therapies, which is particularly important for individualized chemotherapy selection. Four different analytical methods were used in the study to assess the predictive value of the assay. These analyses provide the evidence that this chemoresponse assay is a predictive marker, demonstrating its ability to discern specific therapies that are likely to be more effective among multiple alternatives [50].

As briefly outlined earlier, a chemoresponse assay, such as ChemoFx, is a panel of treatment markers, with the assay result for each treatment evaluated functioning as a distinct marker. When ordering the assay, a physician selects each of the multiple treatments under consideration for a given patient for inclusion in the assay. Clinically validated chemotherapy regimens, consistent with guidelines such as NCCN, comprise the available treatment choices.

Clinical trials designed to evaluate marker or assay efficacy are fundamentally different than trials designed to evaluate drug efficacy. Various different marker trial designs have been extensively studied and reported in the recent literature. However, discussion of effective marker validation trial designs that are appropriate for multiple markers/therapies to be assessed simultaneously (e.g., chemoresponse assays) remains very limited in the current trial design literature. Marker validation trial designs for this type of multiple markers/therapies assay may vary, to some extent, from marker trial designs appropriate for a single molecular biomarker associated with a single therapy (e.g., KRAS/panitumumab, EGFR/erlotinib).

Primarily, three marker study designs have been outlined in the literature for marker validation: enrichment, strategy, and stratified [51–53]. Marker negative patients are excluded in the enrichment design, and thus, it is not applicable to chemoresponse assay evaluation.

Historically, the strategy design, in many ways similar to a standard drug trial design, has been considered the “gold standard” for marker validation as it attempts to emulate what might occur in clinical practice. A variant of the strategy design has been recommended by the American Society of Clinical Oncology (ASCO) and Blue Cross Blue Shield (BCBS) Technical Evaluation Center (TEC) Assessments of validations of chemoresponse assays [54, 55] date back to the mid 1990s. However, multiple recent and updated marker trial design publications, including an evaluation by the Center for Medical Technology Policy (CMTP) in 2013 [56], indicate that the strategy design is less than ideal for marker validation, in that it requires a larger sample size and cannot distinguish between a more effective treatment and marker efficacy, when compared to alternate marker trial designs. Friedlin et al. show that the required sample size for a strategy design can exceed several thousand patients depending on the prevalence of the marker in the study population, presenting a large challenge in a small incidence/prevalence disease like EOC. Further and specific to chemoresponse assays where multiple markers are evaluated simultaneously, the pan-resistant and pan-sensitive patients dilute the ability to assess assay impact on patient outcome. Additionally, overlapping treatments between study arms still further increase the required sample size, rendering the strategy approach essentially pragmatically infeasible. Finally, a potential physician treatment bias or “learning effect” may be associated with the strategy design. The strategy trial design attempted by Cree et al. showed a trend toward improved response rate and PFS in assay-informed patients as compared to those treated with the physician’s empiric choice, but did not achieve statistical significance. Cree et al. asserted that physicians “learned” from the assay-informed arm and began administering treatments similar to those recommended for assay-informed patients to patients in the physician-directed arm as the study progressed. Analysis confirmed this effect; in early physician-choice arm patients, PFS was significantly shorter than that in subsequent year patients [33].

The stratified design has been reported as more efficient, capable of answering the relevant clinical questions, and able to assess both prognostic and predictive marker properties, which is often at issue with evaluations of markers [52, 56, 57]. Friedlin et al. have concluded that trial designs, such as the stratified design that use the marker to guide analysis, but not treatment assignment (i.e., blind or non-interventional designs), are recommended for marker validation [52]. The stratified design has been successfully implemented in multiple clinical validations of clinical guideline recommended markers, including KRAS, EGFR, Oncotype DX^®^ (Genomic Health, Inc., Redwood City, CA, USA), and VeriStrat^®^ (Biodesix, Boulder, CO, USA). The prospective clinical validation trial for the ChemoFx assay required that both the prognostic and predictive properties of the assay be evaluated using an analytical method very similar to the stratified approach [49, 50].

### Clinical utility and economic analysis

An important aspect of clinical utility considers how use of an assay or marker affects patient outcome in terms of treatment selection, survival, and morbidity. Other considerations include the impact of the assay or marker usage on physician treatment plans as well as immediate and downstream healthcare costs [57–59].

To further demonstrate the clinical utility of this chemoresponse assay, we conducted a comparative analysis based on a “two-arm” marker strategy approach. A 192-patient cohort, serving as the assay-informed arm [44], was compared to a non-assay-informed (historical control) arm, comprised of patients treated by non-assay-informed physicians from four large cooperative group drug studies in primary EOC, totaling more than 7,000 patients [2–46]. OS was the primary end point for comparison and analysis. Patient characteristics in both the assay-informed and control arms were similar with the exception that between 11 and 16 % more optimally debulked patients were included in the multiple literature cohorts that comprise the control arm (Table 1). Additionally, while the assay-informed arm and the largest control arm cohort consisted of only advanced stage patients [5, 44], the other three control arm cohorts also included a small portion of earlier stage patients. Based on traditionally accepted adverse clinical variables, a worse prognosis was projected for the assay-informed cohort given the greater proportion of late-stage and sub-optimally debulked patients.

Despite worse prognostic clinical factors, patients in the assay-informed arm experienced a 10 % improvement in median OS compared with the literature-derived control arm (48 vs. 44 months, respectively) [44]. Furthermore, at study completion (6 years follow-up), 39 % of the assay-informed patients were alive compared to 29 % of control arm patients (Table 2).

**Table 2:** Comparison of median OS in control, assay-informed, and assay-informed sensitive cohorts

Survival	du Bois JNCI 2003	Pfisterer JNCI 2006	du Bois JCO 2006	Bookman JCO 2009	Control Arm Average	Assay-Informed Herzog AJOG 2010	Assay-informed Herzog AJOG 2010 Sensitive group
Median OS	44 month	44 month	44 month	44 month	44 month	48 month	72.5 month
1-year	92 %	91 %	90 %	90 %	91 %	85 %	90 %
2-year	74 %	72 %	73 %	75 %	74 %	72 %	86 %
3-year	59 %	57 %	58 %	60 %	59 %	59 %	75 %
4-year	47 %	45 %	46 %	45 %	46 %	51 %	70 %
5-year	38 %	35 %	38 %	35 %	37 %	44 %	60 %
6-year	29 %	NA	28 %	30 %	29 %	39 %	55 %

Annual OS rates in the four cohorts comprising the control arm are based on extrapolation of the published Kaplan–Meier survival curves [2–5, 45]

**Fig. 2:**
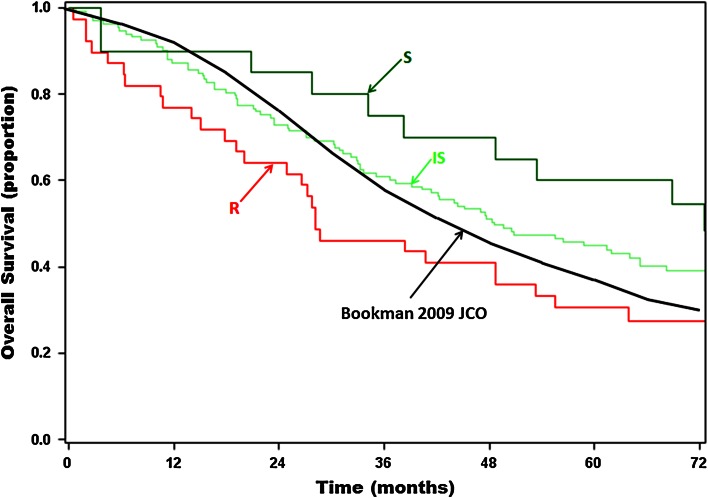
Kaplan–Meier survival curves comparing the control arm cohort [5] (*black*) and the assay-informed arm cohort [45]. Survival curves for the assay-informed cohort were stratified according to assay response category of clinically administered therapy (*S* sensitive, *green*; *IS* intermediate sensitive, *light green*; *R* resistant, *red*)

Median OS for the assay-informed arm, stratified by assay response category, was *S* = 72.5 (*n* = 20), IS = 48.6 (*n* = 133), and *R* = 28.2 months (*n* = 39) (Table 2; Fig. 2), representing a 28.5 month (72.5 vs. 44, 65 %) increased OS for patients treated with assay-S regimens and a 15.8 month (28.2 vs. 44, 36 %) decreased OS for patients treated with assay-R regimens, as compared to the control arm. When comparing annual OS for assay-informed patients treated with an S regimen to control arm patients, 10–25 % more assay-informed patients were living in years 2 thru 6. Notably, 55 % of assay-informed patients treated with an S regimen were alive at year 6 compared to 29 % in the control arm, despite the disparity in adverse clinical factors favoring the control arm (Table 2).

An analysis of survival from patients in the control arm cohorts [2–46] indicates that the various treatment regimens had similar efficacy when therapies were randomly assigned in phase III clinical trials [2–5, 44]. Therefore, even though therapies from the same “pool” of approved and recommended treatment options were administered to patients in the comparative analysis, patients whose treatment was assay-informed had improved survival when compared to patients whose treatment was randomly assigned. Furthermore, in the assay-informed arm, final treatment decisions for patients were made by their physicians, and assays were used to assist treatment selections in some cases and not in others [44]. It is therefore rational to hypothesize that if physicians routinely had chemoresponse information available when choosing chemotherapeutic regimens for patients with ovarian cancer, OS might be further improved.

Average chemotherapy costs for patients with recurrent ovarian cancer treated with or without use of the assay have also been evaluated [60]. Results based on UBS Warburg market share data demonstrated mean costs for chemotherapy treatment were $48,758 for patients treated empirically (no assay), $33,187 for patients with assay results available (65 % adhered to assay results), and $23,986 for patients modeled to have 100 % adherence to assay results. Spanning the median OS of 44 months, the majority of EOC patients experience multiple episodes of disease recurrence [6]. Therefore, treatment costs typically include both surgery and multiple chemotherapy interventions [61]. Considering that assay-informed treatment selection may result in delayed cancer progression and increased OS, if one or more of the multiple chemotherapy interventions were delayed or avoided in assay-informed patients, treatment costs may be reduced by the costs associated with less effective chemotherapy regimens.

Use of chemoresponse assays during primary therapy may help to identify patients with platinum-resistant disease, potentially allowing for consideration of alternate clinically validated [5] or similarly appropriate [65–67] treatments, as well as prognostic stratification of patients in prospective clinical trials and/or modification of primary therapies “off trial” such as the addition of bevacizumab or other targeted therapies to standard carboplatin/paclitaxel treatment [62–64]. Likewise, in the recurrent disease setting where there is no single standard of care, CRSAs may assist oncologists with prioritization of the various single-agent therapies used with or without platinum therapies [54–56]. Additionally, in both primary and recurrent EOC, in the event of a severe drug reaction, physicians may employ this assay to identify an effective (S or IS) therapy with which to replace the toxic agent.

## Conclusions

Despite several years of chemoresponse assay development and clinical experience with these assays, studies have largely been confined to single-institutional, retrospective evaluations. Recent large, prospective, multi-site clinical studies that correlate ChemoFx assay results with overall and progression-free survival in both primary and recurrent ovarian cancers indicate that the assay may offer significant clinical benefit for patients, is predictive of treatment outcomes, and is potentially economically beneficial by reducing the chance that ineffective chemotherapy is administered. This overview supports the inclusion of chemoresponse assay results, along with other clinical factors and biomarkers, to support the individualized selection of effective chemotherapy agents for treatment of patients with ovarian cancer.
